# Ischemic Duration and Frequency Determines AKI-to-CKD Progression Monitored by Dynamic Changes of Tubular Biomarkers in IRI Mice

**DOI:** 10.3389/fphys.2019.00153

**Published:** 2019-02-26

**Authors:** Yang Dong, Qunzi Zhang, Jiejun Wen, Teng Chen, Li He, Yiyun Wang, Jianyong Yin, Rui Wu, Rui Xue, Shiqi Li, Ying Fan, Niansong Wang

**Affiliations:** Department of Nephrology, Shanghai Jiao Tong University Affiliated Sixth People’s Hospital, Shanghai, China

**Keywords:** acute kidney injury, chronic kidney disease, ischemia-reperfusion injury, KIM-1, NGAL

## Abstract

Ischemia reperfusion injury (IRI) is one of the most common causes of acute kidney injury (AKI). However, the pathogenesis and biomarkers predicting the progression of IRI-induced AKI to chronic kidney disease (CKD) remain unclear. A side-by-side comparison between different IRI animal models with variable ischemic duration and episodes was performed. The dynamic changes of KIM-1 and NGAL continuously from AKI to CKD phases were studied as well. Short-term duration of ischemia induced mild renal tubule-interstitial injury which was completely reversed at acute phase of kidney injury, while long-term duration of ischemia caused severe tubular damage, cell apoptosis and inflammatory infiltration at early disease stage, leading to permanent chronic kidney fibrosis at the late stage. Repeated attacks of moderate IRI accelerated the progression of AKI to CKD. Different from serum and urine levels of KIM-1 that increased at acute phase of IRI then declined gradually in chronic phase, NGAL increased continuously during AKI-to-CKD transition. Severity and frequency of ischemia injury determines the progression and outcome of ischemia-induced AKI. Inflammation, apoptosis and fibrogenesis likely participate in the progression of AKI to CKD. Both KIM-1 and NGAL enable noninvasive and early detection of AKI, but NGAL is associated better with the process of AKI-to-CKD progression.

## Introduction

Acute kidney injury is a common syndrome that occurs in approximately 5% of hospitalizations and is often associated with various short- and long-term complications (Uchino et al., 2005). Previously considered to be a benign and reversible syndrome, AKI is now associated with the progression of CKD and ESRD, which lead to approximately 2 million deaths each year globally, and its incidence is growing (Chawla and Kimmel, 2012; Bonventre et al., 2013; Chawla et al., 2014).

Ischemia-reperfusion injury is a pathological process that leads to acute tubulointerstitial injury. IRI may be followed by a repair process that restores the kidney to normal morphology and function if the injury is mild but may also lead to permanent damage, progressive fibrosis and CKD in cases of severe injury (Basile et al., 2012; Lameire et al., 2013). The duration of renal ischemia has been well recognized in clinical studies to be associated with the severity and progression of AKI. Additionally, discharged patients, who suffered from repeated episodes of kidney injury may have an increased risk of progression to CKD. Many studies have used different ischemia/reperfusion (I/R) models to mimic human AKI and study the mechanism of progression from AKI to CKD. However, few studies have provided a side-by-side comparison of AKI prognosis and AKI-to-CKD progression among different I/R animal models with variable ischemia duration and episodes (Ishani et al., 2011; Chawla et al., 2011; Thakar et al., 2011).

In view of various short- and long-term complications of AKI, early detection and treatment are important to improve the prognosis of these patients. Because renal biopsy, the gold standard for the diagnosis of kidney disease, cannot be widely performed in clinical practice due to the contraindication or serious condition of AKI patients, noninvasive biomarkers are urgently needed to predict renal outcomes and identify patients at high risk for CKD progression (Coca and Parikh, 2008). Clinically, a rise in serum creatinine (Scr) is traditionally applied to diagnose AKI. However, during nonsteady-state conditions, Scr-based estimates of glomerular filtration rate (GFR) are inaccurate and insensitive, making assessment of true renal function difficult (Lassnigg et al., 2004; Christensen and Verroust, 2008). Over the past few years, novel noninvasive biomarkers such as kidney injury molecule (KIM)-1 and NGAL have been well considered to be early biomarkers of kidney injury. Recent studies have also speculated KIM-1 and NGAL to be associated with chronic kidney injury (Han et al., 2008; Bolignano et al., 2009; Ko et al., 2010; Bonventre et al., 2013). However, few studies have focused on the dynamic changes in KIM-1 and NGAL levels during AKI-to-CKD progression.

In the present study, we performed a detailed side-by-side comparison among different I/R animal models with variable ischemia duration and episodes and studied the dynamic changes in KIM-1, NGAL, and Scr from AKI to CKD phases. We hope to provide a better understanding of these I/R models and their relevance to human AKI, and to help researchers and clinicians better monitor AKI progression.

## Materials and Methods

### Animal Models

Male C57BL/6J mice weighing 22–25 g were purchased from Shanghai Science Academy Animal Center (Shanghai, China) (Supplementary Table S1), housed under a constant 12-h light–dark cycle at a temperature between 21°C and 23°C and allowed free access to food and water. Mice were subjected to acute ischemic kidney injury induced by unilateral ischemia-reperfusion (UIRI). Briefly, mice were given general anesthesia of an intraperitoneal injection of a mixture of ketamine hydrochloride (100 mg/kg body weight) and xylazine hydrochloride (10 mg/kg body weight). The left kidney was exposed through a midline abdominal incision, followed by the induction of ischemia in the kidney with the placement of an atraumatic microaneurysm clamp (Fine Science Tools, Cambridge, United Kingdom) on the left renal pedicle. After a predetermined time of ischemia, the clamps were removed, and the reperfusion of the kidney was visually confirmed by a color change from dark purple back to red. Sham-operated mice were subjected to a similar surgical procedure without clamping of the left renal pedicles. Throughout the surgery, body temperature was maintained at 37 ± 0.3°C with a temperature-controlled heating system (Alcott Biotech Co., Ltd, Shanghai, China), and the mouse body was covered with gauze soaked in warm water to preserve moisture. Due to the compensatory effect of the right kidney, the UIRI model allowed the study of a large range of kidney injury severity. To evaluate the function of the injured kidney, the healthy contralateral kidney was removed 24h before the collection of the blood sample for Scr measurement (Supplementary Figure S1A). All animal experiments were approved by the Laboratory Animal Care and Ethical Committee of Shanghai Jiao Tong University Affiliated Sixth People’s Hospital.

### Blood and Urine Biochemical Measurements

Serum creatinine was determined using a Quantichrom assay kit (BioAssay Systems, Hayward, CA, United States) according to the manufacturer’s protocol. Urinary kidney injury molecule 1 (uKIM-1) and urinary neutrophil gelatinase-associated lipocalin (uNGAL), as well as their counterparts serum kidney injury molecule 1 (sKIM-1) and neutrophil gelatinase-associated lipocalin (sNGAL), were measured using enzyme-linked immunosorbent assay (ELISA) kits (R&D Systems, MN, United States).

### Histology and Quantitative Assessment of Tubular Injury and Fibrosis

Paraformaldehyde-fixed kidneys were dehydrated in ethanol and embedded in paraffin. Then, the kidney tissue blocks were sectioned to a thickness of 3 μm for light microscopy. Hematoxylin-eosin (HE) and Masson trichrome staining were performed to assess histological injury and fibrosis. Tubular injury was scored semiquantitatively by a blinded observer who examined at least 20 cortical fields (×200 magnification) of periodic acid-Schiff (PAS)-stained sections. Tubular injury was defined as tubular dilation, tubular atrophy, tubular cast formation, sloughing of tubular epithelial cells or loss of the brush border and thickening of the tubular basement membrane using the following scoring system: Score 0: no tubular injury; Score 1: <10% of tubules injured; Score 2: 10–25% of tubules injured; Score 3: 25–50% of tubules injured; Score 4: 50–74% of tubules injured; Score 5: >75% of tubules injured (Chen et al., 2013). Quantification of fibrosis was performed by measuring the presence of interstitial fibrosis in Masson trichrome-stained sections from each kidney. Briefly, digital images of at least 20 cortical fields ( × 200 magnification) were examined by an experienced pathologist in a blinded manner and scored using the following scale: Score 0: no evidence of interstitial fibrosis; Score 1: <25% involvement; Score 2: 25 to 50% involvement: Score 3: >50% involvement (Ots et al., 1998).

### Immunohistochemistry Staining

Paraformaldehyde-fixed, paraffin-embedded 3-μm-thick sections were deparaffinized and rehydrated. After antigen retrieval, sections were incubated overnight with antibodies against α-SMA, collagen 1, Ly6g, Ki-67 (Abcam, United States) and F4/80 (ABD Serotec, United Kingdom) at 4°C (Supplementary Table S3), followed by biotinylated secondary antibody (Dako, Carpinteria, CA, United States) for 60 min at 37°C. Then, 3,3-diaminobenzidine was used as the chromogen. Finally, the slides were counterstained with hematoxylin and mounted after dehydration. The staining was semiquantitatively scored separately on a scale of 0–4 in a blinded manner by an experienced pathologist (Score 0: absence of specific staining; Score 1: <25% area has specific staining; Score 2: 25 to 50%; Score 3: 50 to 75%; Score 4: >75% ) (Fan et al., 2015). Ly6g, F4/80 and Ki-67-positive cells were quantified by counting the number of stained cells per high-power field (hpf) (×400) with 10 hpfs included in each slide.

### Western Blot Analysis

Tissue was lysed with RIPA buffer containing protease and phosphatase inhibitor cocktail. The α-SMA antibody (Abcam, United States) was used for immunoblot analysis. We repeated each Western blot analysis using protein from three separate experiments. The specific protein bands were scanned using the Western Blotting Detection System (BIO-RAD). The area of each band was analyzed using the National Institutes of Health image software (ImageJ).

### Real-Time PCR Quantification

Total RNA was extracted from kidney samples using Trizol (Invitrogen, Carlsbad, CA, United States). RNA was reversed-transcribed into c-DNA using Superscript III First-Strand Synthesis SuperMix (Invitrogen, Carlsbad, CA, United States). Real-time PCR was performed with the Step One Plus System (Applied Biosystems, Foster City, CA, United States) using SYBR Green Master Mix (Qiagen). Using the 2-ΔΔCt method, relative quantification of the gene expression was determined (Supplementary Table S2). Gene expression was normalized to GAPDH.

### Apoptosis Assessment

TUNEL staining for cell apoptosis in renal tissues was employed to evaluate the extent of apoptosis using an ApopTag^®^ Red *In Situ* Apoptosis Detection Kit (Millipore, United States). The number of apoptotic cells with red fluorescent nuclear staining was calculated using fluorescence microscopy. Apoptotic nuclei were quantified in ten hpfs (x400) per slide.

### Statistical Analysis

Data are expressed as the mean ± standard error (SEM). The two-sided unpaired *t*-test was used to analyze data between two groups after determination of data distributions and variance. ANOVA with Bonferroni correction was used when more than two groups were present. Spearman correlation analysis was used to analyze correlations. GraphPad Prism software, version 6.0 was used for statistical analyses. *P*-values were two-sided, and *P* < 0.05 was considered statistically significant.

## Results

### Relationship Between Ischemia Duration and Severity of Acute Tubule-Interstitial Injury in IRI Mice

**FIGURE 1:**
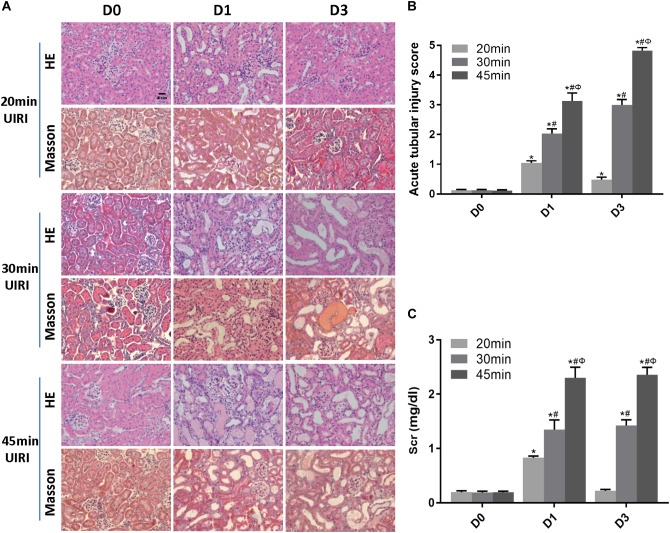
Relationship between ischemia duration and severity of acute tubule-interstitial injury. **(A)** HE and Masson staining of kidneys during the acute phase of kidney injury (original magnification × 400; Scale bar, 20 μm). **(B)** Acute tubular injury score of mouse kidneys during the acute phase of kidney injury. **(C)** Changes in Scr levels in mice during the acute phase of kidney injury. Data are presented as the means ± SEM of four experiments. *n* = 6; ^∗^*P* < 0.05 vs. the control group. #*P* < 0.05 vs. the 20 min-ischemia group. Φ*P* < 0.05 vs. the 30 min-ischemia group.

We first examined the effect of ischemia duration on kidney injury in UIRI mice. Mice subjected to 20 min-UIRI showed mild renal tubular injury at day 1 post-ischemia, which reversed to normal at day 3 and was completely recovered at day 7 (Figure 1A,B). However, 30 min-UIRI caused severe tubular interstitial damage characterized by tubular lumen dilatation, cellular detachment from tubular basement membranes, tubular cast formation and focal inflammatory cell infiltration in the interstitium, which got even worse in the 45 min-UIRI mouse group (Figure 1A,B). Glomerular injury was absent in all conditions. Consistent with the histological observations, the Scr level was significantly elevated at day 1 after 20 min of UIRI but returned to baseline 3 days after IRI. However, in the 30 and 45 min-UIRI mouse groups, the Scr level was increased at day 1 post-ischemia and was even higher at day 3 (Figure 1C). These data suggest an ischemia time-dependent change in kidney injury in these IRI mice.

### A Long Ischemia Duration Induced Kidney Fibrosis and AKI-to-CKD Transition in IRI Mice

**FIGURE 2:**
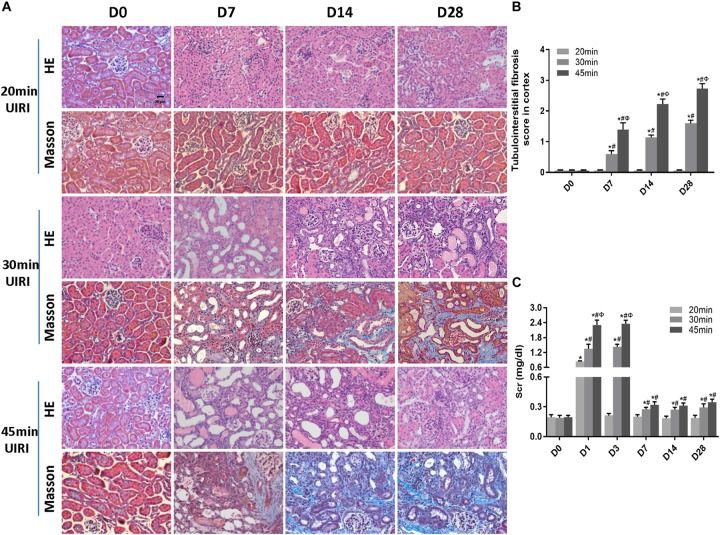
A long ischemia duration induced AKI-to-CKD transition in IRI mice. **(A)** HE and Masson staining of kidneys during the chronic phase of kidney injury (original magnification × 400; Scale bar, 20 μm). **(B)** Tubulointerstitial fibrosis scores of mouse kidneys during the chronic phase of kidney injury. **(C)** Changes in Scr levels in mice in 28 days post ischemia. Data are presented as the means ± SEM of four experiments. *n* = 6; ^∗^*P* < 0.05 vs. the control group. #*P* < 0.05 vs. the 20 min-ischemia group. Φ*P* < 0.05 vs. the 30 min-ischemia group.

**FIGURE 3:**
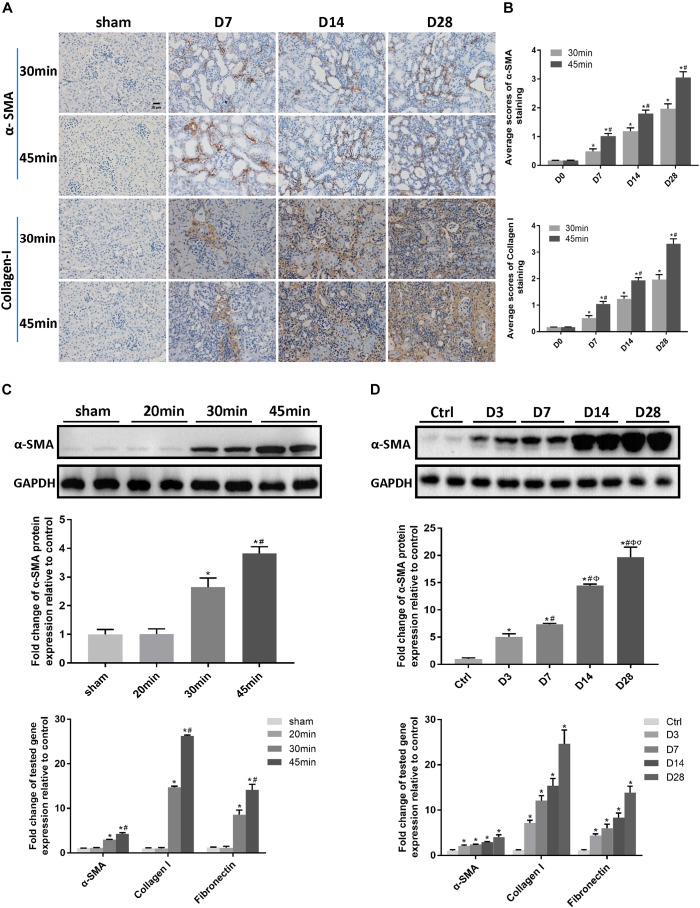
A long ischemia duration induced kidney fibrosis in IRI mice. **(A)** Immunohistochemical staining of α-SMA and collagen I in kidneys, with representative pictures shown (original magnification × 400; Scale bar, 20μm). **(B)** Semiquantitative data of α-SMA and collagen I staining in different groups of mice. ^∗^*P* < 0.05 vs. the control group. #*P* < 0.05 vs. the 30-min ischemia group. **(C)** Protein and mRNA expression of fibrosis markers in the kidney tissue lysates of mice subjected to different durations of ischemia at day 28 post-operation. ^∗^*P* < 0.05 vs. the sham control group. #*P* < 0.05 vs. the 30-min ischemia group. **(D)** Protein and mRNA expression of fibrosis markers in the kidney tissue lysates of mice subjected to 45 min-IRI at different time points post ischemia. ^∗^*P* < 0.05 vs. the baseline control group. #*P* < 0.05 vs. Day 3. Φ*P* < 0.05 vs. Day 7. σ*P* < 0.05 vs. Day 14. All data above are presented as the means ± SEM of four experiments. *n* = 6.

We next investigated the effect of a long ischemia duration on the progression of kidney injury in IRI mice. Kidney mass at day 28 post-ischemia was significantly reduced in mice subjected to 30 or 45 min-IRI (Supplementary Figures S2A,B). Histologically, H&E and Masson’s trichrome staining showed features of chronic injury in kidney sections of the 30 min-UIRI mouse group characterized by significant tubular dilatation, casts or intraluminal debris, kidney interstitial fibrosis and diffused infiltration of inflammation starting at day 7 post-ischemia, which were even worse in the 45 min-UIRI mouse group (Figure 2A). Fibrosis scores showed an increase in fibrosis from day 7 to day 28 post-ischemia, and a much higher score was observed in the 45-min UIRI mouse group than in the 30-min UIRI group (Figure 2B). To confirm the effect of long-term ischemia on fibrosis in IRI kidney, we found that the expression of fibrosis markers (α-SMA, collagen I and fibronectin) was progressively elevated from day 7 to day 28 post-ischemia in an ischemia time-dependent manner, as measured by immunohistochemistry, western blot, and real-time PCR (Figure 3A–D). As shown in Figure 2C, Scr was elevated significantly at day 1 and day 3 post-ischemia, though it decreased beginning on day 7, the level of Scr was still increased at day 7, 14 and 28 post IRI as compared to the baseline (*P* < 0.05). Our data confirmed that severe ischemic kidney injury caused renal fibrosis in IRI mice.

### Elevated Apoptosis and Inflammation Were Associated With IRI-Induced AKI Severity and the AKI-to-CKD Transition

**FIGURE 4:**
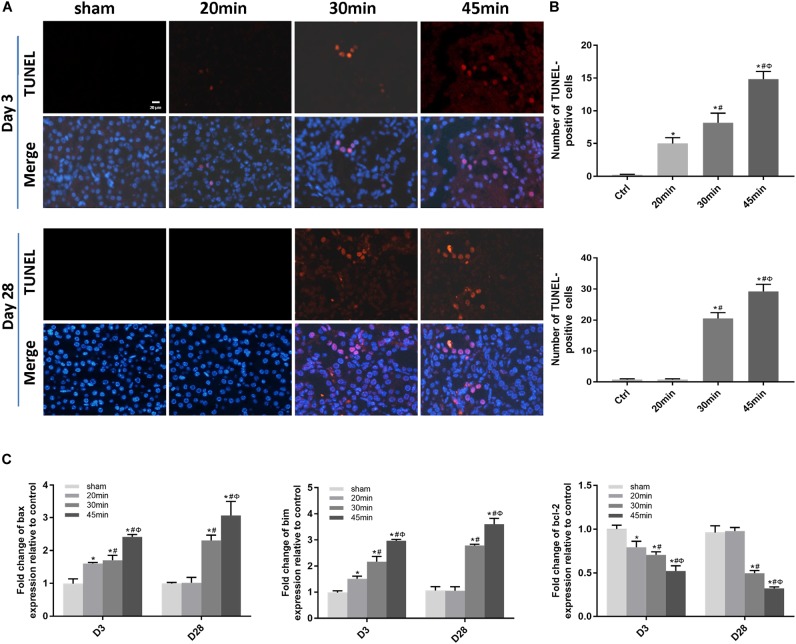
Elevated apoptosis was associated with IRI-induced AKI severity and the AKI-to-CKD progression. **(A)** TUNEL staining to measure the apoptosis of renal tubular epithelial cells in each mouse group at days 3 and 28 post ischemia (original magnification × 400; Scale bar, 20 μm). **(B)** Quantitative analysis of the number of apoptotic cells in each group. **(C)** Expression of proapoptotic genes (Bax and Bim) and the antiapoptotic gene bcl-2 was measured by real-time PCR. Data are presented as the means ± SEM of four experiments. *n* = 6; ^∗^*P* < 0.05 vs. the control group. #*P* < 0.05 vs. the 20-min ischemia group. Φ*P* < 0.05 vs. the 30 min-ischemia group.

**FIGURE 5:**
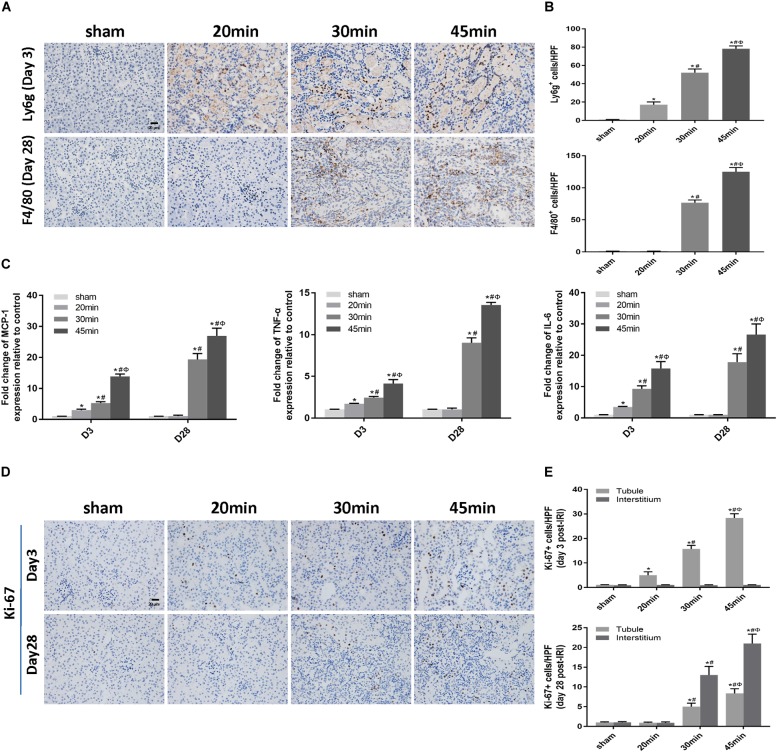
Elevated inflammation was associated with IRI-induced AKI severity and the AKI-to-CKD transition. **(A)** Immunohistochemical staining of Ly6g (a neutrophil marker) at day 3 and F4/80 (a macrophage marker) at day 28 post ischemia in kidneys subjected to different durations of ischemia (original magnification × 400; Scale bar, 20 μm). **(B)** Semiquantitative data of Ly6g and F4/80 staining in different groups of mice. **(C)** Expression of inflammatory mediators (MCP1, TNF-α, and IL-6) was measured by real-time PCR. **(D)** Immunohistochemical staining of Ki-67 (a marker of proliferation) at day 3 and day 28 post ischemia in kidneys subjected to different durations of ischemia (original magnification × 400; Scale bar, 20 μm). **(E)** Semiquantitative data of Ki-67 staining in different groups of mice. T, tubule; I, interstitium. Data are presented as the means ± SEM of four experiments. *n* = 6; ^∗^*P* < 0.05 vs. the control group. #*P* < 0.05 vs. the 20 min-ischemia group. Φ*P* < 0.05 vs. the 30 min-ischemia group.

We then performed TUNEL staining to investigate whether apoptosis was involved in IRI-induced AKI. Apoptotic cells were detected not only at the acute phase of AKI (day 3 post-ischemia) but also during the AKI-to-CKD transition (day 28 post-ischemia) in the tubular compartment of 30 min-UIRI mice and were more prominent in 45 min-UIRI mice (Figure 4A,B). Upregulation of proapoptotic genes (bax and bim) and downregulation of the antiapoptotic gene bcl-2 were also detected by real-time PCR (Figure 4C). We further examined the infiltration of inflammatory cells and the expression of inflammatory mediators in the IRI kidneys. Immunostaining showed significantly more neutrophil accumulation by Ly6g staining in the 30 min-UIRI group than in the 20 min-UIRI mice on day 3, and the expression of Ly6g was more pronounced in the kidney sections of the 45 min-UIRI group (Figure 5A,B). Gene expression of inflammatory mediators, monocyte chemoattractant protein 1 (MCP1), tumor necrosis factor (TNF)-α and interleukin (IL)-6, by real-time PCR also showed an ischemia time-dependent increase in the ischemic kidneys at the early stage of AKI (Figure 5C). As an indicator of chronic inflammation, significantly greater infiltration of macrophages was found in 30 and 45 min-UIRI than in the 20 min-UIRI mice at day 28 post-ischemia, associated with increased gene expression of MCP1, TNF-α, and IL-6 (Figure 5A–C). To obtain a better understanding of the repair process after IRI, we stained for Ki-67, a well-known marker for cell proliferation. Increased Ki-67 expression was found in renal tubular epithelial cells at the acute phase of IRI and in both tubular and interstitial cells at the later stage when there was an AKI-to-CKD transition (Figure 5D,E). These results suggest that elevated apoptosis, inflammation and an imbalance of renal cell proliferation/apoptosis in response to time-dependent ischemic injury may contribute to AKI severity and the AKI-to-CKD transition.

### Repeated Episodes of Moderate IRI Accelerated Kidney Injury in Mice

**FIGURE 6:**
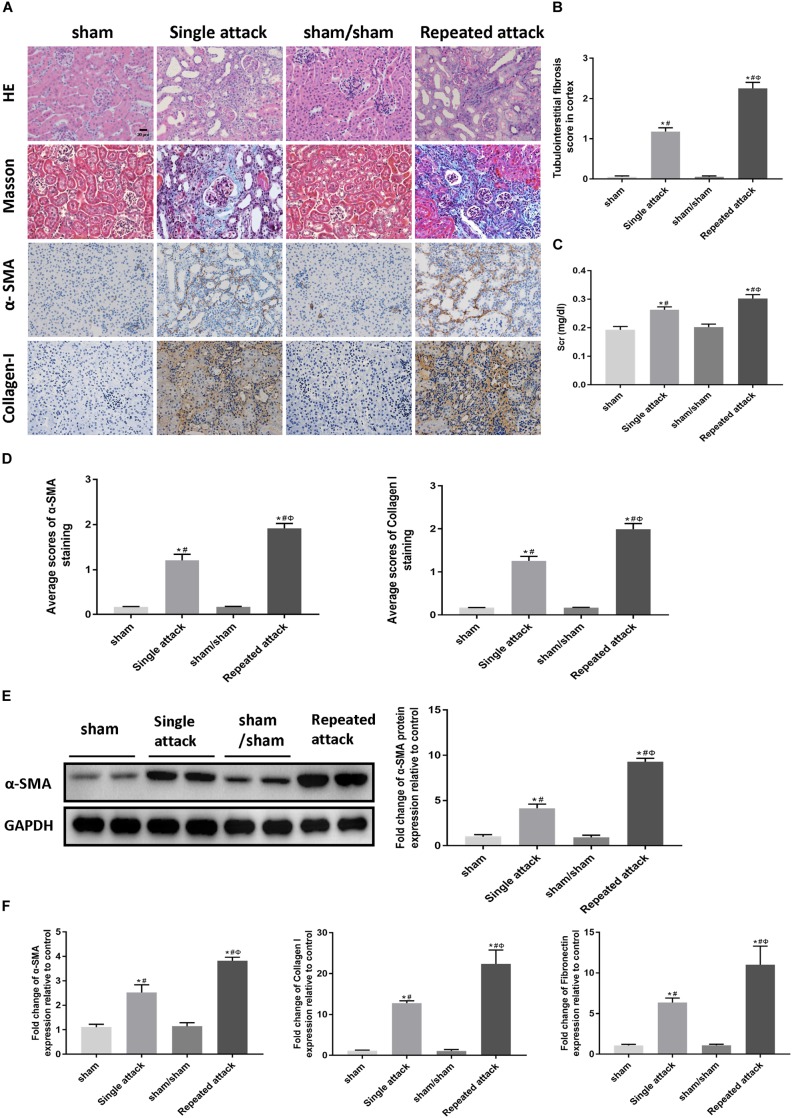
Repeated episodes of moderate IRI accelerated kidney injury in mice. **(A)** HE and Masson’s trichrome staining as well as immunohistochemical staining of α-SMA and collagen I in kidneys at day 14 after the first renal ischemia attack are shown (original magnification × 400; Scale bar, 20 μm). **(B)** Tubulointerstitial fibrosis score of mouse kidneys at day 14 after the first renal ischemia attack. **(C)** Changes in Scr levels in mice at day 14. **(D)** Semiquantitative data of α-SMA and collagen I staining in different groups of mice. **(E)** Western blot analysis of α-SMA in the kidney tissue lysates of mice in each group. The densitometry analyses of Western blot are shown. **(F)** Effect of the frequency of ischemia attack on the mRNA expression of fibrosis markers in kidney tissue lysates. Data are presented as the means ± SEM of four experiments. *n* = 6; ^∗^*P* < 0.05 vs. the sham control group. #*P* < 0.05 vs. the sham/sham group. Φ*P* < 0.05 vs. the single-attack group.

To examine whether repeated episodes of IRI accelerate kidney injury and kidney fibrosis, mice were subjected to a single 30 min-UIRI attack, and then a repeated episode of 30 min-UIRI was performed after a 7 day-interval (Supplementary Figure S1B). Data showed that a single episode of 30 min-UIRI resulted in tubular dilatation, focal infiltration of inflammatory cells and moderate interstitial fibrosis at day 14 post-ischemia. However, the lesions in the repeated-attack group were more serious (Figure 6A,B). Consistently, Scr levels in mice subjected to repeated 30 min-UIRI attack were significantly higher than those in mice subjected to a single attack (Figure 6C). Assessment of renal fibrosis markers (α-SMA, collagen I and fibronectin) showed worse renal fibrosis scores in repeated-attacked IRI mice compared to those in single-attack mice, as measured by immunostaining, western blot and real-time PCR (Figure 6A,D–F). These findings indicate that repeated episode of I/R attack accelerates kidney injury and the AKI-to-CKD progression in IRI mice.

### Increased Apoptosis and Inflammation Were Associated With the Acceleration of the AKI-to-CKD Transition in Mice Exposed to Repeated IRI

**FIGURE 7:**
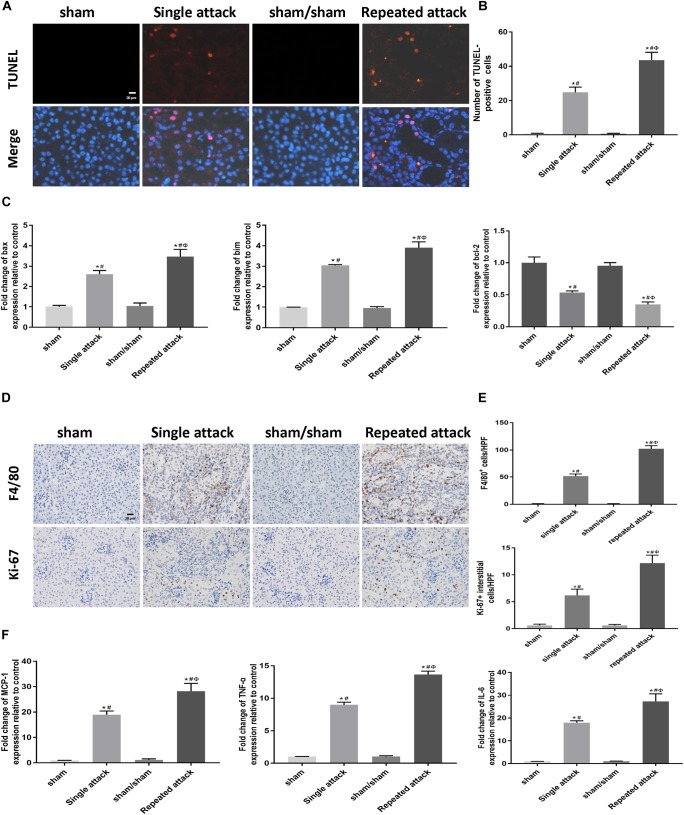
Increased apoptosis and inflammation were associated with the acceleration of the AKI-to-CKD transition in mice exposed to repeated IRI attacks. **(A)** TUNEL staining to measure the apoptosis of renal tubular epithelial cells in kidneys at day 14 after the first renal ischemia attack (original magnification × 400; Scale bar, 20 μm). **(B)** Quantitative analysis of the number of apoptotic cells in each group. **(C)** Expression of proapoptotic genes (bax and bim) and the antiapoptotic gene bcl-2 were measured by real-time PCR. **(D)** Immunohistochemical staining of F4/80 (a macrophage marker) and Ki-67 (a marker of proliferation) at day 14 after the first renal ischemia attack (original magnification × 400; Scale bar, 20 μm). **(E)** Semiquantitative data of F4/80 and Ki-67 staining in different groups of mice. **(F)** Expression of inflammatory mediators (MCP1, TNF-α, and IL-6) was measured by real-time PCR. Data are presented as the means ± SEM of four experiments. *n* = 6; ^∗^*P* < 0.05 vs. the sham control group. #*P* < 0.05 vs. the sham/sham group. Φ*P* < 0.05 vs. the single-attack group.

To examine whether apoptosis and inflammation were also associated with the repeated IRI-induced acceleration of AKI-to-CKD transition, we studied cell apoptosis status as well as the expression of inflammatory markers in the IRI kidneys. TUNEL staining showed that the number of tubular apoptotic cells increased along with the repeated IRI attack, followed by upregulation of proapoptotic genes (bax and bim) and downregulation of bcl-2 (Figure 7A–C). Immunostaining revealed significantly more infiltration of macrophages in kidney sections of repeated-attack mice than in those of the single-attack mice (Figure 7D,E). Repeated-attack mice showed significantly higher MCP1, TNF-α, and IL-6 expression than single-attack mice, as assessed by real-time PCR (Figure 7F). A significant increase in interstitial cell proliferation was also detected in kidney sections of the repeated IRI group by Ki-67 staining (Figure 7D,E). These data suggest that increased apoptosis, inflammation and interstitial cell proliferation might be involved in the acceleration of the AKI-to-CKD transition in mice exposed to repeated IRI attacks.

### Dynamic Changes in KIM-1 and NGAL From AKI to CKD Phases in IRI Mice With Different Ischemia Durations and Episodes

To explore the role of novel noninvasive biomarkers in the early detection of AKI, we measured the levels of KIM-1 and NGAL in the serum and urine of 10 and 15 min-UIRI mice. Our data showed that, although there was no change in morphology or Scr levels (Supplementary Figures S3A,B), the levels of KIM-1 and NGAL in the blood and urine were significantly higher in the 10 and 15 min-UIRI mouse group than in the control group (Supplementary Figures S3C–F), suggesting that KIM-1 and NGAL are more sensitive than Scr in detecting early injury.

**FIGURE 8:**
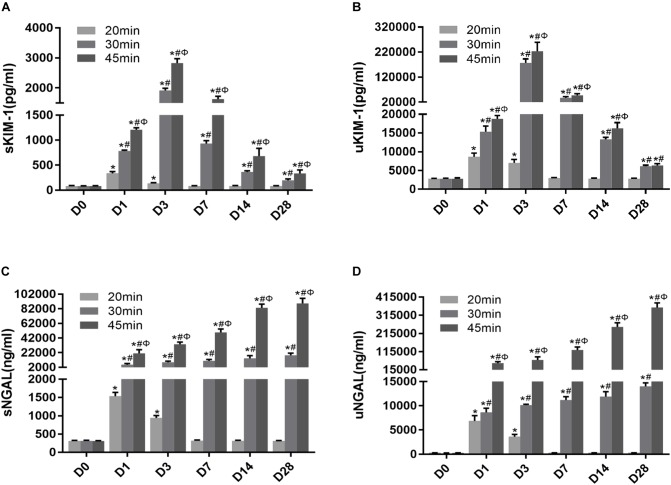
Novel AKI biomarkers KIM-1 and NGAL enabled the noninvasive and early detection of AKI. **(A–D)** Tubular injury biomarker changes in mice subjected to different durations of ischemia at 28 days post ischemia: **(A)** serum KIM-1, **(B)** urinary KIM-1, **(C)** serum NGAL, **(D)** urinary NGAL. Data are presented as the means ± SEM of four experiments. *n* = 6; ^∗^*P* < 0.05 vs. the control group. #*P* < 0.05 vs. the 20-min ischemia group. Φ*P* < 0.05 vs. the 30-min ischemia group.

**FIGURE 9:**
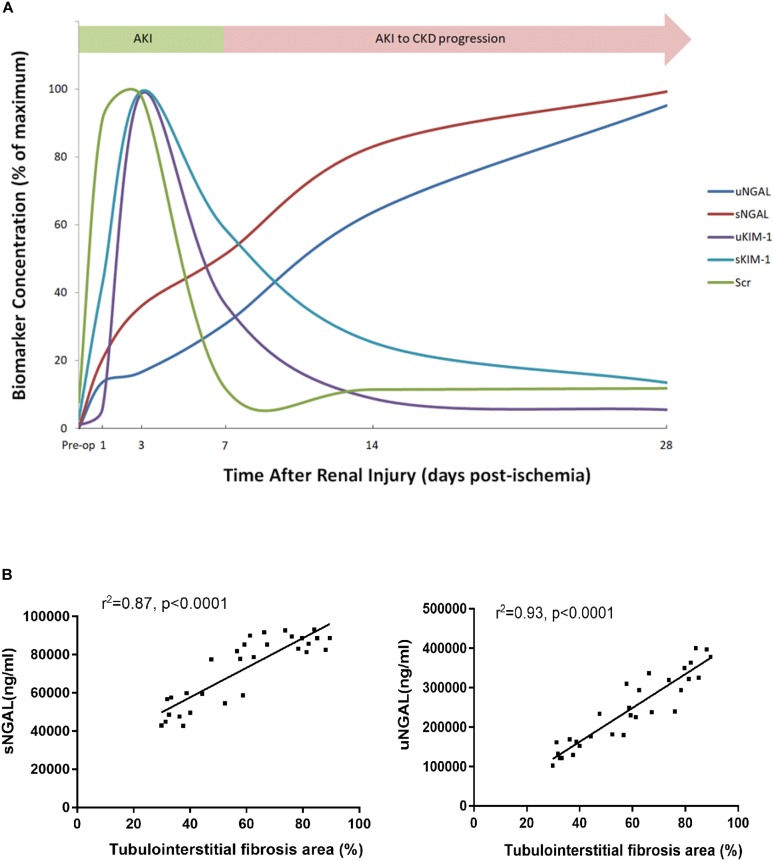
NGAL was better associated with AKI-to-CKD progression. **(A)** The time course of changes in Scr levels and biomarker concentration after 45 min of UIRI. To create this plot, the level at the time point with the highest mean value was set to 100%, and the levels at all other time points were converted to a percentage of this maximum. **(B)** Correlations were analyzed between sNGAL and the extent of tubulointerstitial fibrosis (*r* = 0.87, *P* < 0.0001) and uNGAL and the extent of tubulointerstitial fibrosis (*r* = 0.93, *P* < 0.0001). sNGAL: serum NGAL, uNGAL: urinary NGAL.

To determine whether KIM-1 and NGAL could be used to assess the long-term effect of ischemia-induced kidney injury on the AKI-to-CKD transition, the time course of changes in the serum and urine concentrations of KIM-1 and NGAL were examined on days 1, 3, 7, 14 and 28 post-ischemia in different mice group (Figure 8A–D). Further more, the dynamic changes in KIM-1, NGAL and Scr levels in 45 min-IRI mice were plotted against time, with the level at the time point with the highest mean value set to 100%, and the levels at all other time points converted to a percentage of this maximum. As shown in Figure 9A, both serum and urine levels of creatinine and KIM-1 were transiently increased at the acute stage of kidney injury, peaking at day 3, decreasing significantly beginning on day 7 and remaining slightly higher than baseline at day 28 post-ischemia. Notably, the serum and urine levels of NGAL progressively increased from the initiation of IRI until the chronic phase of the disease, which remained substantially elevated at day 28 post-ischemia, suggesting that NGAL might be a better biomarker to dynamically monitor AKI-to-CKD progression (Figure 9A). We also found a positive association between sNGAL/uNGAL level and tubulointerstitial fibrosis by histological quantification in UIRI mice with AKI-to-CKD progression (*r* = 0.87, 0.93, *P* < 0.01) (Figure 9B). In addition, both KIM-1 and NGAL levels were much higher in mice exposed to repeated episodes of 30 min-UIRI than in mice exposed to single 30 min-UIRI attack (Supplementary Figures S4A–D). Taken together, these data indicate that both KIM-1 and NGAL enable the early detection of kidney injury, but NGAL might be a better biomarker for sustained kidney injury and AKI-to-CKD progression.

## Discussion

Ischemia reperfusion injury is one of the most common causes of AKI in clinical practice, which is associated with high medical cost, increased length of hospital stay and in-hospital mortality (Uchino et al., 2005). In contrast to the traditional view that the renal function of AKI patients tends to recover completely, an increasing number of clinical studies have indicated that survivors of AKI may have a considerable risk of progressing to CKD (Chawla and Kimmel, 2012; Bonventre et al., 2013; Chawla et al., 2014). To clarify to what extent the severity of tubule injury was associated with renal prognosis, we examined UIRI mouse models with different durations of ischemia. Our data suggested that AKI severity and the subsequent AKI-to-CKD progression are dependent on ischemia duration in this model, which is in accordance with previous studies showing that, although multiple clinical factors such as medical condition, advanced age and genetic diversity play a role, AKI severity seems to be the most significant risk factor for poor long-term outcome (Ishani et al., 2011; Chawla et al., 2011, 2014; Chawla and Kimmel, 2012).

Discharged AKI patients are known to be susceptible to a repeated episode of kidney attack, which may increase the risk of rehospitalization and accelerate the progression of kidney disease. In a hospital-based cohort of patients with diabetes mellitus, Thakar et al. (2011) found that AKI increases the risk of advanced CKD in diabetic patients and that each episode of AKI doubles the risk. Data from another community-based cohort of patients with CKD suggests that an episode of superimposed acute renal failure was associated with a high risk of irreversible renal dysfunction and aggravated the progression to ESRD (Hsu et al., 2009). Our data demonstrated that a repeated AKI episode on a kidney previously lesioned with 30 min-UIRI accelerated the AKI-to-CKD transition. This finding was consistent with the results of a previous study showing that repeated proximal tubule injuries induced by diphtheria toxin administration could cause sustained interstitial fibrosis (Takaori et al., 2016).

Thus far, the underlying mechanism of why a prolonged ischemia duration or repeated episodes of IRI lead to a greater susceptibility to severe kidney injury and AKI-to-CKD transition is still incompletely understood. Apoptosis is a key characteristic of AKI. Recently, Dong et al. found that conditional Bax deletion specifically from proximal tubules attenuated renal tubular cell apoptosis and ameliorated IRI in Bax-deficient mouse models (Wei et al., 2013). In another study, Padanilam et al. indicated that p53 has profound effects on tubular cell apoptosis after IRI. The absence of p53 in the proximal tubule significantly preserves renal function and reduces kidney damage (Ying et al., 2014). Our data revealed increased tubular cell apoptosis, elevated proapoptotic gene expression and decreased antiapoptotic genes in the IRI kidneys at both the acute and chronic phases, suggesting that mice suffering from severe AKI underwent maladaptive repair process of cell apoptosis, potentially leading to kidney fibrosis and irreversible chronic injury. Proinflammatory leukocytes have also been speculated to play important roles in the pathogenesis of IRI (Jang and Rabb, 2015). In our research, infiltration of neutrophils and macrophages was found to increase at the early and late stages of IRI in an ischemia time-dependent manner, which is consistent with a previous study showing that macrophage depletion attenuated the gene expression of inflammatory and profibrotic cytokines in an IRI mouse model (Lech et al., 2014). It is known that Ki-67 is a nuclear non-histone protein that is presented at low levels in quiescent cells, but increased in proliferating cells, especially in the G2, M, and latter half of the S phase (Cuylen et al., 2016; Sun and Kaufman, 2018). Ki-67 is used as a marker to measure tubular regeneration and renal repair after AKI (Lazzeri et al., 2018; Zhou et al., 2018). Ki-67+ proliferating in tubular and interstitial cells has been found to be evident in CKD animal models as well (Bijkerk et al., 2016; Raman et al., 2017). In our study, we also found increased Ki-67 expression in renal tubular epithelial cells at acute phase of IRI, whereas it occurred in both tubular and interstitial cells at the later stage when there was an AKI-to-CKD transition. 30 min of ischemia time appears to be a cut off since interstitial Ki-67 alterations occur only when ischemia time was ≥30 min, indicating the ischemic time-dependent injury in tubular interstitium. A possible explanation for these observations could be that defects in the progression of the cell cycle after injury may switch tubular cells to a pro-fibrotic phenotype, thus promoting a fibrogenic response (Yang et al., 2010). Therefore, severe AKI could result in incomplete repair and persistent tubulointerstitial inflammation, with proliferation of fibroblasts and excessive deposition of extracellular matrix, leading to CKD and even progression to ESRD.

The early identification and initiation of treatment in AKI patients who are at risk of developing progressive CKD is known to substantially improve the prognosis of the disease. Over the past few years, KIM-1 and NGAL have been recognized as early biomarkers of AKI (Mishra et al., 2003; Ichimura et al., 2004; Han et al., 2008, 2009). In our study, KIM-1 and NGAL were shown to be more sensitive markers than Scr in mild ischemic AKI mice and were able to detect renal dysfunction at a very early phase, even before the appearance of kidney histological injuries. Recent studies have also speculated KIM-1 and NGAL to be associated with chronic kidney injury (Bolignano et al., 2009; Ko et al., 2010; Bonventre et al., 2013). However, few studies have focused on the temporal profile of KIM-1 and NGAL levels during AKI-to-CKD progression. Here, for the first time, we examined the concentration of KIM-1 and NGAL at different time points during disease progression in IRI mouse models to evaluate whether these two tubular biomarkers could provide a dynamic monitoring of AKI-to-CKD progression. Our data showed that serum and urine levels of KIM-1 peaked at the acute phase but decreased gradually afterward, while NGAL increased continuously from the initiation of AKI to the chronic phase of the disease, indicating NGAL might be a better biomarker than KIM-1 in monitoring progression of AKI to CKD. This was consistent with the findings in a matched case-control study of 143 CKD patients suggesting that higher NGAL levels, but not KIM-1 levels, were associated with incident CKD stage 3 (Bhavsar et al., 2012).

NGAL, a 25-kDa protein of the lipocalin superfamily, is expressed at low levels in normal human tissues and is rapidly released from renal tubular cells after various injuring stimuli, which represents a novel, sensitive, specific biomarker for early detection of AKI (Mishra et al., 2003; Han et al., 2008, 2009). In our study, blood and urine levels of NGAL increased transiently in the reversible AKI model, whereas NGAL levels increased substantially and continuously during the AKI-to-CKD transition in the progressive IRI mice model. Another transcriptional study also demonstrated the gene expression of NGAL remained in high level during AKI-to-CKD progression in IRI mice (Ko et al., 2010). Clinically, NGAL may be a marker of CKD progression as well (Mitsnefes et al., 2007; Bolignano et al., 2008, 2009; Malyszko et al., 2008; Carrero and Stenvinkel, 2011; Bhavsar et al., 2012; AvciÇiçek et al., 2016). Bolignano D et al. measured NGAL in patients affected by non-advanced and non-terminal CKD and found that both serum and urinary levels of NGAL were increased in CKD patients and were inversely correlated with eGFR (Bolignano et al., 2009). Another cohort study of 163 patients with CKD stages I–V and 82 healthy volunteers also demonstrated that serum NGAL levels are significantly higher in CKD group than healthy group (AvciÇiçek et al., 2016). In this study, to further test if NGAL might be an ideal biomarker to monitor AKI-to-CKD progression, we found a positive correlation between the concentration of NGAL in body fluid and the extent of tubulointerstitial fibrosis in kidney sections of IRI mice. Therefore, we suggest that progressively increasing levels of NGAL might reflect sustained kidney injury and AKI-to-CKD progression.

## Conclusion

In conclusion, in the current study, we established UIRI mouse models to mimic diverse clinical outcomes of ischemia-induced kidney injury in AKI patients: reversible AKI, AKI-to-CKD progression and recurrent AKI. We carefully assessed how the severity and frequency of ischemia injury determined the progression and outcome of ischemia-induced AKI. Inflammation, cell apoptosis and fibrogenesis are likely the underlying mechanisms contributing to the AKI-to-CKD transition. Both serum and urine levels of KIM-1 and NGAL enable the noninvasive and early detection of AKI. However, NGAL better reflect AKI-to-CKD transition. We believe that our study is the first to perform a detailed side-by-side comparison of different IRI animal models with variable ischemia durations and frequencies. Our findings will be helpful for scientists to select an adequate IRI model for studying different pathological processes related to AKI. We also believe that our study is the first to provide a temporal profile of KIM-1 and NGAL from the acute phase to the chronic phase in an IRI mouse model, which may help researchers and clinicians better monitor AKI progression.

## Author Contributions

YF, NW, and YD designed the research project. YD, QZ, JW, TC, LH, RW, JY, and RX performed the experiments. YD, YF, YW, and SL analyzed the data. YD and YF drafted the manuscript. YF and NW edited and revised the manuscript and approved the final version of the manuscript.

## Conflict of Interest Statement

The authors declare that the research was conducted in the absence of any commercial or financial relationships that could be construed as a potential conflict of interest.

## Abbreviations

α-SMA: α-smooth muscle actin
AKI: acute kidney injury
CKD: chronic kidney disease
ESRD: end-stage renal disease
GAPDH: glyceraldehyde 3-phosphate dehydrogenase
IRI: ischemia-reperfusion injury
KIM-1: kidney injury molecule 1
Ly6g: lymphocyte antigen 6 complex locus G6D
NGAL: neutrophil gelatinase-associated lipocalin

## References

[B1] AvciÇiçek E. Rota S. Dursun B. Kavalci E. (2016). Evaluation of serum NGAL and hepcidin levels in chronic kidney disease patients. *Ren. Fail.* 38 35–39. 10.3109/0886022X.2015.1107823 26627016

[B2] Basile D. P. Anderson M. D. Sutton T. A. (2012). Pathophysiology of acute kidney injury. *Compr. Physiol.* 2 1303–1353. 10.1002/cphy.c110041 23798302 PMC3919808

[B3] Bhavsar N. A. Köttgen A. Coresh J. Astor B. C. (2012). Neutrophil gelatinase-associated lipocalin (NGAL) and kidney injury molecule 1 (KIM-1) as predictors of incident CKD stage 3: the atherosclerosis risk in communities (ARIC) study. *Am. J. Kidney Dis.* 60 233–240. 10.1053/j.ajkd.2012.02.336 22542304 PMC3399971

[B4] Bijkerk R. de Bruin R. G. van Solingen C. van Gils J. M. Duijs J. M. van der Veer E. P. (2016). Silencing of microRNA-132 reduces renal fibrosis by selectively inhibiting myofibroblast proliferation. *Kidney Int.* 89 1268–1280. 10.1016/j.kint.2016.01.029 27165825

[B5] Bolignano D. Bolignano D. Lacquaniti A. Coppolino G. Campo S. Arena A. (2008). Neutrophil gelatinase-associated lipocalin reflects the severity of renal impairment in subjects affected by chronic kidney disease. *Kidney Blood Press. Res.* 31 255–258. 10.1159/000143726 18600028

[B6] Bolignano D. Lacquaniti A. Coppolino G. Donato V. Campo S. Fazio M. R. (2009). Neutrophil gelatinase-associated lipocalin (NGAL) and progression of chronic kidney disease. *Clin. J. Am. Soc. Nephrol.* 4 337–344. 10.2215/CJN.03530708 19176795 PMC2637601

[B7] Bonventre J. V. Basile D. Liu K. D. McKay D. Molitoris B. A. Nath K. A. (2013). AKI: a path forward. *Clin. J. Am. Soc. Nephrol.* 8 1606–1608. 10.2215/CJN.06040613 23868899 PMC3805072

[B8] Carrero J. J. Stenvinkel P. (2011). Predialysis chronic kidney disease in 2010: novel targets for slowing CKD progression. *Nat. Rev. Nephrol.* 7 65–66. 10.1038/nrneph.2010.177 21278712

[B9] Chawla L. S. Amdur R. L. Amodeo S. Kimmel P. L. Palant C. E. (2011). The severity of acute kidney injury predicts progression to chronic kidney disease. *Kidney Int.* 79 1361–1369. 10.1038/ki.2011.42 21430640 PMC3257034

[B10] Chawla L. S. Eggers P. W. Star R. A. Kimmel P. L. (2014). Acute kidney injury and chronic kidney disease as interconnected syndromes. *N. Engl. J. Med.* 371 58–66. 10.1056/NEJMra1214243 24988558 PMC9720902

[B11] Chawla L. S. Kimmel P. L. (2012). Acute kidney injury and chronic kidney disease: an integrated clinical syndrome. *Kidney Int.* 82 516–524. 10.1038/ki.2012.208 22673882

[B12] Chen J. Chen J. K. Conway E. M. Harris R. C. (2013). Survivin mediates renal proximal tubule recovery from AKI. *J. Am. Soc. Nephrol.* 24 2023–2033. 10.1681/ASN.2013010076 23949800 PMC3839548

[B13] Christensen E. I Verroust P. J. (2008). Interstitial fibrosis: tubular hypothesis versus glomerular hypothesis. *Kidney Int.* 74 1233–1236. 10.1038/ki.2008.421 18974759

[B14] Coca S. G. Parikh C. R. (2008). Urinary biomarkers for acute kidney injury: perspectives on translation. *Clin. J. Am. Soc. Nephrol.* 3 481–490. 10.2215/CJN.03520807 18256377 PMC6631074

[B15] Cuylen S. Blaukopf C. Politi A. Z. Müller-Reichert T. Neumann B. Poser I. (2016). Ki-67 acts as a biological surfactant to disperse mitotic chromosomes. *Nature* 535 308–312. 10.1038/nature18610 27362226 PMC4947524

[B16] Fan Y. Xiao W. Li Z. Li X. Chuang P. Y. Jim B. (2015). RTN1 mediates progression of kidney disease by inducing ER stress. *Nat. Commun.* 6:7841. 10.1038/ncomms8841 26227493 PMC4532799

[B17] Han W. K. Wagener G. Zhu Y. Wang S. Lee H. T. (2009). Urinary biomarkers in the early detection of acute kidney injury after cardiac surgery. *Clin. J. Am. Soc. Nephrol.* 4 873–882. 10.2215/CJN.04810908 19406962 PMC2676184

[B18] Han W. K. Waikar S. S. Johnson A. Betensky R. A. Dent C. L. Devarajan P. (2008). Urinary biomarkers in the early diagnosis of acute kidney injury. *Kidney Int.* 73 863–869. 10.1038/sj.ki.5002715 18059454 PMC2586909

[B19] Hsu C. Y. Chertow G. M. McCulloch C. E. Fan D. Ordoñez J. D. Go A. S. (2009). Nonrecovery of kidney function and death after acute or chronic renal failure. *Clin. J. Am. Soc. Nephrol.* 4 891–898. 10.2215/CJN.05571008 19406959 PMC2676192

[B20] Ichimura T. Hung C. C. Yang S. A. Stevens J. L. Bonventre J. V. (2004). Kidney injury molecule-1 a tissue and urinary biomarker for nephrotoxicant-induced renal injury. *Am. J. Physiol. Renal. Physiol.* 286 F552–F563. 10.1152/ajprenal.00285.2002 14600030

[B21] Ishani A. Nelson D. Clothier B. Schult T. Nugent S. Greer N. (2011). The magnitude of acute serum creatinine increase after cardiac surgery and the risk of chronic kidney disease, progression of kidney disease, and death. *Arch. Intern. Med.* 171 226–233. 10.1001/archinternmed.2010.514 21325112

[B22] Jang H. R. Rabb H. (2015). Immune cells in experimental acute kidney injury. *Nat. Rev. Nephrol.* 11 88–101. 10.1038/nrneph.2014.180 25331787

[B23] Ko G. J. Grigoryev D. N. Linfert D. Jang H. R. Watkins T. Cheadle C. (2010). Transcriptional analysis of kidneys during repair from AKI reveals possible roles for NGAL and KIM-1 as biomarkers of AKI-to-CKD transition. *Am. J. Physiol. Renal. Physiol.* 298 F1472–F1483. 10.1152/ajprenal.00619.2009 20181666

[B24] Lameire N. H. Bagga A. Cruz D. Maeseneer J. D. Endre Z. Kellum J. A. (2013). Acute kidney injury: an increasing global concern. *Lancet* 382 170–179. 10.1016/S0140-6736(13)60647-9 23727171

[B25] Lassnigg A. Schmidlin D. Mouhieddine M. Bachmann L. M. Druml W. Bauer P. (2004). Minimal changes of serum creatinine predict prognosis in patients after cardiothoracic surgery: a prospective cohort study. *J. Am. Soc. Nephrol.* 15 1597–1605. 10.1097/01.ASN.0000130340.93930.DD 15153571

[B26] Lazzeri E. Angelotti M. L. Peired A. Conte C. Marschner J. A. Maggi L. (2018). Endocycle-related tubular cell hypertrophy and progenitor proliferation recover renal function after acute kidney injury. *Nat.Commun.* 9:1344. 10.1038/s41467-018-03753-4 29632300 PMC5890293

[B27] Lech M. Gröbmayr R. Ryu M. Lorenz G. Hartter I. Mulay S. R. (2014). Macrophage phenotype controls long-term AKI outcomes–kidney regeneration versus atrophy. *J. Am. Soc. Nephrol.* 25 292–304. 10.1681/ASN.2013020152 24309188 PMC3904561

[B28] Malyszko J. Bachorzewska-Gajewska H. Sitniewska E. Malyszko J. S. Poniatowski B. Dobrzycki S. (2008). Serum neutrophil gelatinase-associated lipocalin as a marker of renal function in non-diabetic patients with stage 2–4 chronic kidney disease. *Ren. Fail.* 30 625–628. 10.1080/08860220802134607 18661413

[B29] Mishra J. Ma Q. Prada A. Mitsnefes M. Zahedi K. Yang J. (2003). Identification of neutrophil gelatinase-associated lipocalin as a novel early urinary biomarker for ischemic renal injury. *J. Am. Soc. Nephrol.* 14 2534–2543. 10.1097/01.ASN.0000088027.54400.C6 14514731

[B30] Mitsnefes M. M. Kathman T. S. Mishra J. Kartal J. Khoury P. R. Nickolas T. L. (2007). Serum neutrophil gelatinase-associated lipocalin as a marker of renal function in children with chronic kidney disease. *Pediatr. Nephrol.* 22 101–108. 10.1007/s00467-006-0244-x 17072653

[B31] Ots M. Mackenzie H. S. Troy J. L. Rennke H. G. Brenner B. M. (1998). Effects of combination therapy with enalapril and losartan on the rate of progression of renal injury in rats with 5/6 renal mass ablation. *J. Am. Soc. Nephrol.* 9 224–230. 10.1089/end.1998.12.81 9527398

[B32] Raman A. Reif G. A. Dai Y. Khanna A. Li X. Astleford L. (2017). Integrin-linked kinase signaling promotes cyst growth and fibrosis in polycystic kidney disease. *J. Am. Soc. Nephrol.* 28 2708–2719. 10.1681/ASN.2016111235 28522687 PMC5576939

[B33] Sun X. Kaufman P. D. (2018). Ki-67: more than a proliferation marker. *Chromosoma* 127 175–186. 10.1007/s00412-018-0659-8 29322240 PMC5945335

[B34] Takaori K. Nakamura J. Yamamoto S. Nakata H. Sato Y. Takase M. (2016). Severity and frequency of proximal tubule injury determines renal prognosis. *J. Am. Soc. Nephrol.* 27 2393–2406. 10.1681/ASN.2015060647 26701981 PMC4978049

[B35] Thakar C. V. Christianson A. Himmelfarb J. Leonard A. C. (2011). Acute kidney injury episodes and chronic kidney disease risk in diabetes mellitus. *Clin. J. Am. Soc. Nephrol.* 6 2567–2572. 10.2215/CJN.01120211 21903988 PMC3359576

[B36] Uchino S. Kellum J. A. Bellomo R. Doig G. S. Morimatsu H. Morgera S. (2005). Acute renal failure in critically ill patients: a multinational, multicenter study. *JAMA* 294 813–818. 10.1001/jama.294.7.813 16106006

[B37] Wei Q. Dong G. Chen J. K. Ramesh G. Dong Z. (2013). Bax and bak have critical roles in ischemic acute kidney injury in global and proximal tubulespecific knockout mouse models. *Kidney Int.* 84 138–148. 10.1038/ki.2013.68 23466994 PMC3686831

[B38] Yang L. Besschetnova T. Y. Brooks C. R. Shah J. V. Bonventre J. V. (2010). Epithelial cell cycle arrest in G2/M mediates kidney fibrosis after injury. *Nat. Med.* 16 535–543. 10.1038/nm.2144 20436483 PMC3928013

[B39] Ying Y. Kim J. Westphal S. N. Long K. E. Padanilam B. J. (2014). Targeted deletion of p53 in the proximal tubule prevents ischemic renal injury. *J. Am. Soc. Nephrol.* 25 2707–2716. 10.1681/ASN.2013121270 24854277 PMC4243356

[B40] Zhou D. Fu H. Xiao L. Mo H. Zhuo H. Tian X. (2018). Fibroblast-Specificβ-Catenin Signaling Dictates the Outcome of AKI. *J. Am. Soc. Nephrol.* 29 1257–1271. 10.1681/ASN.2017080903 29343518 PMC5875957

