# Supplementary material for: Ischemic Duration and Frequency Determines AKI-to-CKD Progression Monitored by Dynamic Changes of Tubular Biomarkers in IRI Mice
Source: Front Physiol. 2019 Feb 26;10:153. doi: 10.3389/fphys.2019.00153 (PMC6401609; doi:10.3389/fphys.2019.00153)
Supplement: Supplementary file 1 [file Data_Sheet_1.PDF]

Supplementary Material

Table S1: Comparison of renal function among HN rats and Febuxostat treated groups at different time points.

	**sham**	**10min-UIRI**	**15min-UIRI**	**20min-UIRI**	**30min-UIRI**	**45min-UIRI**	**Single attack**	**Repeated attack**
Body weights (g)	23.19±0.99	23.39±0.86	23.64±0.85	23.20±0.80	23.43±1.03	23.20±0.73	23.60±0.96	23.32±0.83

*p<0.05 for comparisons between the experimental and sham groups

Table S2: Primer sequences used in real-time PCR

Target gene	Primer sequences
α-SMA	Forward 5’ CCCTGAAGAGCATCCGACA 3’
	Reverse 5’ CTCCAGAGTCCAGCACAATACC 3’
collagen I	Forward 5’ GAGGGCGAGTGCTGTGCT 3’
	Reverse 5’ GTCCAGGGATGCCATCTCG 3’
fibronectin	Forward 5’ CAAAGATGACAAGGAAAGTGCC 3’
	Reverse 5’ GCCGCAACTACTGTGATTCG 3’
GADPH	Forward 5’ TGTTCCTACCCCCAATGTGTC 3’
	Reverse 5’ TGAAGTCGCAGGAGACAACC 3’
bim	Forward 5’ CTGAGTGTGACAGAGAAGGTGGAC 3’
	Reverse 5’ CGGTTCTGTCTGTAGGGAGGTAG 3’
bax	Forward 5’ GGTTGCCCTCTTCTACTTTGC 3’
	Reverse 5’ GCCGCTCACGGAGGAAG 3’
bcl-2	Forward 5’ CTACCGTCGTGACTTCGCAG 3’
	Reverse 5’ CCCACCGAACTCAAAGAAGG 3’
MCP-1	Forward 5’ GCTGACCCCAAGAAGGAATG 3’
	Reverse 5’ TTGAGGTGGTTGTGGAAAAGG 3’
TNF-α	Forward 5’ CCCTCCAGAAAAGACACCATG 3’
	Reverse 5’ CACCCCGAAGTTCAGTAGACAG 3’
IL-6	Forward 5’ GGGACTGATGCTGGTGACAAC 3’
	Reverse 5’ CAACTCTTTTCTCATTTCCACGA 3’

Table S3: Antibodies used in Western blot and Immunohistochemical staining

Antibodies	Company	Cat. No	Species	Molecular Weight
α-SMA	ABCAM	ab5694	Rabbit	42kDa
collagen1	ABCAM	ab34710	Rabbit	139kDa
ki-67	ABCAM	Ab1666	Rabbit	345-395kDa
ly6g	ABCAM	Ab25377	Rabbit	25kDa
F4/80	ABD Serotec	MCA497GA	Rat	160KDa

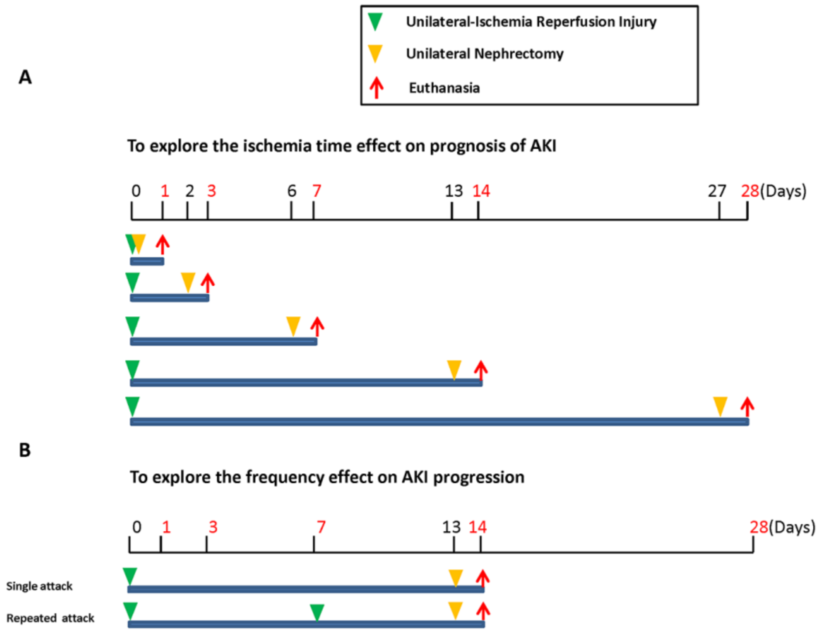

Figure S1. Diagram for the experimental design. (**A**) Experimental design for the study about effect of ischemia duration on the kidney injury prognosis. The green and orange arrowheads indicate the time point of unilateral ischemia–reperfusion injury (UIRI) and unilateral nephrectomy (UNx) respectively. The red arrows indicate the time point when the mice were euthanized. Different durations of ischemia were performed on mice. (**B**) Experimental design for the study about effect of ischemia injury frequency on the AKI progression. The green and orange arrowheads indicate the time point of UIRI and UNx respectively. The red arrows indicate the time point when the mice were euthanized. In this section, only 30 min-UIRI was performed on mice.

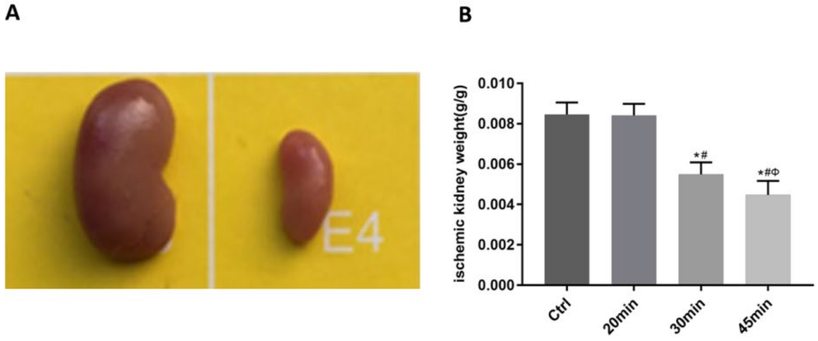

Figure S2. Long-term ischemia induces a significant renal mass reduction in mice. (**A**) Gross structural changes are observed between ischemic kidneys (right) and contralateral kidneys (left): the ischemic kidney is shrunken in size. (**B**) Ischemic kidney weight at euthanasia in mice subjected to different ischemia durations at day 28 post-ischemia. Kidney weights are corrected for body weight. Data are presented as the means ±SEM of four experiments. n = 6. * P<0.05 vs. the sham control group. # P<0.05 vs. the 20 min-ischemia group. ΦP<0.05 vs. the 30 min-ischemia group.

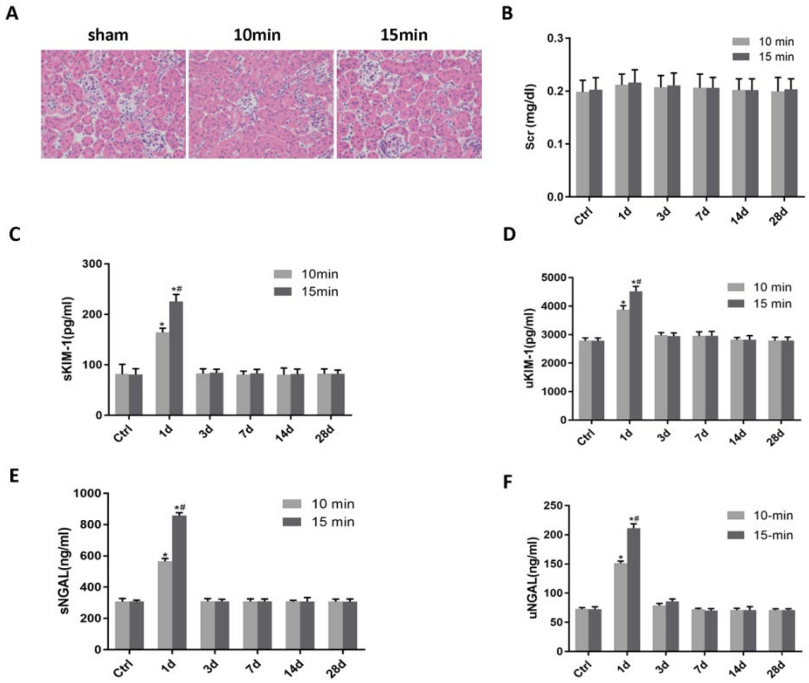

Figure S3. Levels of tubular injury biomarker as well as histological and functional changes in mice subjected to 10 and 15-min UIRI. (**A**) HE staining of kidney sections at day 1 post different durations of ischemia (original magnification ×400). (**B**) Changes in serum creatinine of mice at day 1 post-ischemia. (**C-F**) serum KIM-1 (**C**) , urinary KIM-1 (**D**) , serum NGAL (**E**) and urinary NGAL (**F**). Data are presented as the means ±SEM of four experiments. n = 6. * P<0.05 vs. the control group. # P<0.05 vs. the 10 min-ischemia group.

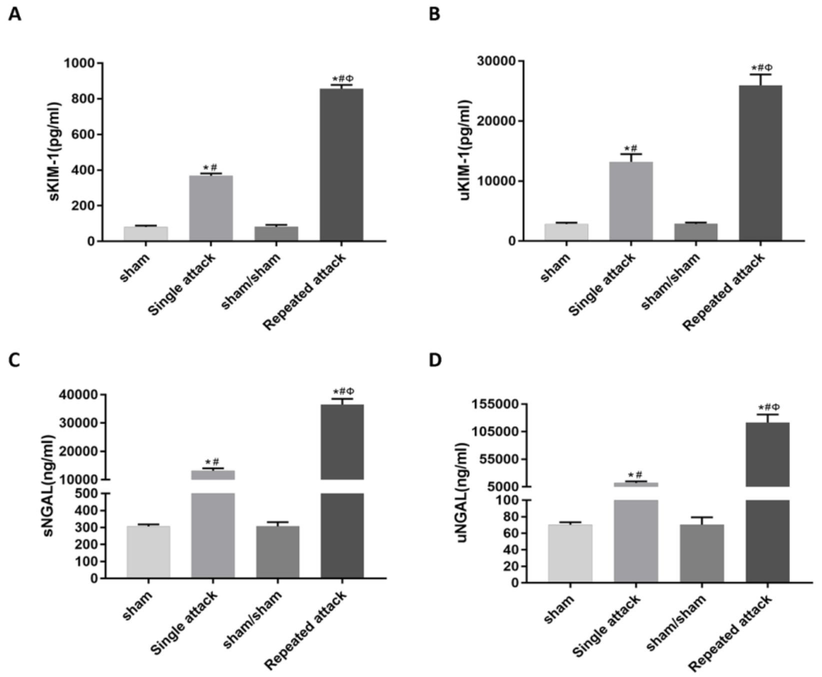

Figure S4. Levels of tubular injury biomarker in mice subjected to single or repeated moderate IRI attack. (**A**) serum KIM-1, (**B**) urinary KIM-1, (**C**) serum NGAL, (**D**) urinary NGAL. The data are presented as the means ±SEM of four experiments. n = 6. * $P<0.05$ vs. the sham control group. # $P<0.05$ vs. the sham/sham group. Φ$P<0.05$ vs. the single attack group.
